# One 3D VOI-based deep learning radiomics strategy, clinical model and radiologists for predicting lymph node metastases in pancreatic ductal adenocarcinoma based on multiphasic contrast-enhanced computer tomography

**DOI:** 10.3389/fonc.2022.990156

**Published:** 2022-09-09

**Authors:** Hongfan Liao, Junjun Yang, Yongmei Li, Hongwei Liang, Junyong Ye, Yanbing Liu

**Affiliations:** ^1^ College of Medical Informatics, Chongqing Medical University, Chongqing, China; ^2^ Key Laboratory of Optoelectronic Technology and Systems of the Ministry of Education, Chongqing University, Chongqing, China; ^3^ Department of Radiology, The First Affiliated Hospital of Chongqing Medical University, Chongqing, China

**Keywords:** pancreatic ductal adenocarcinoma, lymph node metastases, deep learning, radiomics, contrast-enhanced computer tomography

## Abstract

**Purpose:**

We designed to construct one 3D VOI-based deep learning radiomics strategy for identifying lymph node metastases (LNM) in pancreatic ductal adenocarcinoma on the basis of multiphasic contrast-enhanced computer tomography and to assist clinical decision-making.

**Methods:**

This retrospective research enrolled 139 PDAC patients undergoing pre-operative arterial phase and venous phase scanning examination between 2015 and 2021. A primary group (training group and validation group) and an independent test group were divided. The DLR strategy included three sections. (1) Residual network three dimensional-18 (Resnet 3D-18) architecture was constructed for deep learning feature extraction. (2) Least absolute shrinkage and selection operator model was used for feature selection. (3) Fully connected network served as the classifier. The DLR strategy was applied for constructing different 3D CNN models using 5-fold cross-validation. Radiomics scores (Rad score) were calculated for distinguishing the statistical difference between negative and positive lymph nodes. A clinical model was constructed by combining significantly different clinical variables using univariate and multivariable logistic regression. The manifestation of two radiologists was detected for comparing with computer-developed models. Receiver operating characteristic curves, the area under the curve, accuracy, precision, recall, and F1 score were used for evaluating model performance.

**Results:**

A total of 45, 49, and 59 deep learning features were selected *via* LASSO model. No matter in which 3D CNN model, Rad score demonstrated the deep learning features were significantly different between non-LNM and LNM groups. The AP+VP DLR model yielded the best performance in predicting status of lymph node in PDAC with an AUC of 0.995 (95% CI:0.989-1.000) in training group; an AUC of 0.940 (95% CI:0.910-0.971) in validation group; and an AUC of 0.949 (95% CI:0.914-0.984) in test group. The clinical model enrolled the histological grade, CA19-9 level and CT-reported tumor size. The AP+VP DLR model outperformed AP DLR model, VP DLR model, clinical model, and two radiologists.

**Conclusions:**

The AP+VP DLR model based on Resnet 3D-18 demonstrated excellent ability for identifying LNM in PDAC, which could act as a non-invasive and accurate guide for clinical therapeutic strategies. This 3D CNN model combined with 3D tumor segmentation technology is labor-saving, promising, and effective.

## Introduction

Pancreatic ductal adenocarcinoma (PDAC), as the 2^nd^ principal criminal of global cancer death rate by 2030, is notorious due to early metastasis and latent occult, with the 5-year long-term survival remaining merely 7%–8% (1, 2). Early surgical excision is the sole radical therapy protocol available for PDAC patients. The occurrence of lymph node metastases (LNM) in PDAC is well known to be a vital hazard of PDAC, manifesting poor prognosis after surgical resection (3). National comprehensive cancer network (NCCN) guidelines reported PDAC patients with positive status of lymph nodes should receive pre-operative neo-adjuvant treatment, and survival time could obviously improve after surgical resection (4–6). Thus, accurately and timely prediction of LNM prior to treatment is significant for providing the best treatment strategy for PDAC patients. Currently, contrast-enhanced computer tomography (CECT) is regarded as the dominating examination mechanics in the recognition of lymph nodes (7–9), but its overall accuracy is far from satisfactory owing to it is easily influenced by inflammatory hyperplasia or secondary biliary obstruction (10, 11). Other imaging examinations, such as magnetic resonance imaging or positron emission tomography, were regarded as supplementary predictive tools, whereas manifested inconspicuous advantage (12). Moreover, endoscopic ultrasonography-guided fine needle aspiration, which could obtain one piece of specimen, is highly invasive and has the risk of interventional complications, such as pancreatitis and pancreatic fistula (13, 14). Thus, one precise and noninvasive diagnosis strategy is needed.

Recently, computational aid in diagnosis (CAD) developed a state-of-the-art technology in medical images research area that could convert macroscopic images to thousands of underlyingly quantitative features, thereby improving diagnostic performance and assist in clinical decision-making (15, 16). Currently, most radiomics studies used traditional machine learning methods like support vector machine (SVM) to solve clinical problems and manifest moderate results (17–20). However, traditional machine learning approaches exist two primary shortcomings as yet. On the one hand, it is up to manual segmentation as gold standard, and this work requires experienced radiologists to spend much time and energy. On the other hand, it extracted only handcrafted features that are relatively low-level and concrete (21). However, deep learning, as an emerging informatic technology, automatically extracts the higher-level features from medical images without human intervention (22, 23), which precisely preserves the objectivity and nature of the data, achieving quite outstanding performance in various medical tasks. Convolutional neural networks (CNN), as one most representative deep learning architecture, have been extensively applied for image analysis and outperforms traditional machine learning methods in the aspect of reproducibility and repeatability (24, 25). Previous radiomics studies (26, 27) using two-dimensional (2D) CNN models, most focused on 2D ROI-based segmentation *via* inputting slices one by one, only capturing spatial correlation while leaving the rich three-dimensional (3D) context information unexploited. Therefore, choosing 3D CNN model based on 3D volume of interest (VOI) structure for regarding tumor as an interactively whole entirety is consequential.

Currently, most radiomics studies aiming at differentiation of LNM in PDAC were based on traditional machine learning methods with tedious procedure and unsatisfactory generalization ability (28–32), and the utilization of 3D VOI combined with 3D CNN on the basis of multiphasic CECT for identifying LNM in PDAC is rarely been reported. Therefore, we designed to construct three different Residual network 3D-18 (Resnet 3D-18) CNN models including AP DLR model, VP DLR model, and AP+VP DLR model for this classification task, not only avoiding cost-timely manual segmentation, but also protecting the integrity of tumor structure. We have confidence that our findings could not only provide an outstanding predictive strategy for PDAC patients with LNM but also lead one 3D VOI-based 3D CNN technology to exploit more advanced research.

## Materials and methods

### Patients

**Figure 1:**
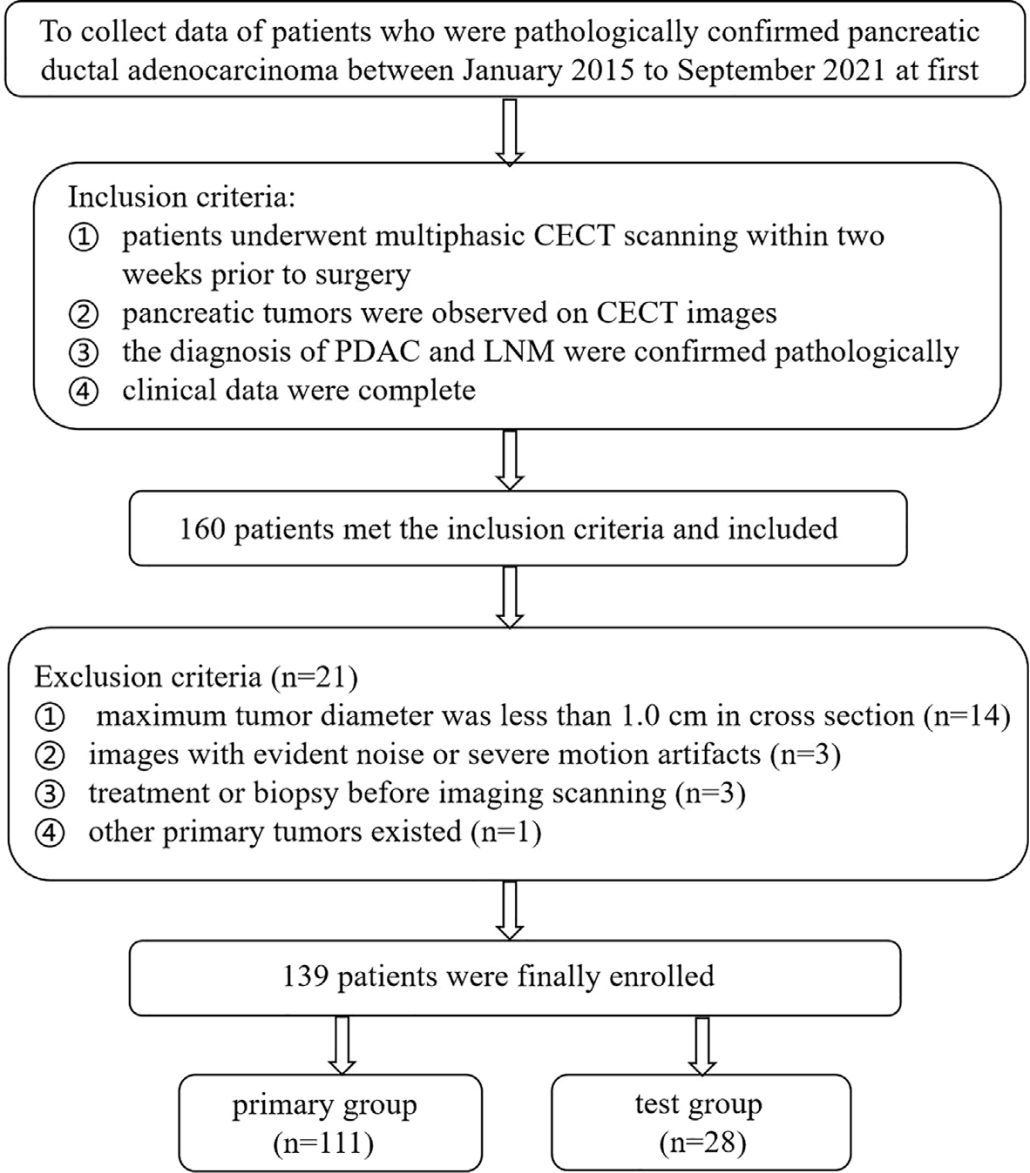
Flow chart of patient’s selection.

The ethics committee of the First Affiliated Hospital of Chongqing Medical University approved this study (No:2022-63), and the demand for informed consent was exempted. Primary PDAC patients underwent surgery resection with standard regional lymph node dissection from January 2015 to September 2021 were collected. The enrolled criteria were as follows: (1) patients underwent arterial phase (AP) and venous phase (VP) scanning within 2 weeks prior to surgery; (2) pancreatic tumors were observed on CECT images; (3) the diagnosis of PDAC and LNM were confirmed pathologically; and (4) clinical data were completed. The exclusion criteria were as follows: (1) tumor diameter was at least 1.0 cm; (2) images with evident noise or severe motion artifacts; (3) treatment or biopsy before imaging scanning; and (4) other primary tumors existed. The patients were partitioned into a primary group (training group and validation group) and a test group at a proportion of 8:2 using random sampling. The patient selection flowchart is described in **Figure 1**.

### Image acquisition

One 128-slice multidetector-row CT scanner (SOMATOM Definition Flash, Siemens Healthineers) was performed. The CT scanning parameters were set as follows: 120 kV; 300 mA; 0.7 pitch; collimation, 128 × 0.6 mm; beam collimation, 160×0.5 mm; matrix, 512×512; and gantry rotation time, 0.5 s. Non-ionic contrast agent (Ultravist 370, Bayer Schering Pharma) was injected into the antecubital vein using a pump injector (Medrad Mark V plus, Bayer). The injection dose was 1.2 ml/kg and the flow rate was 3.5 ml/s. Then, normal saline of 40 ml was injected to flush the tube. Unenhanced phase was scanned first. Approximately 15 s after the abdominal aorta reaching 100 HU, AP scanning was performed, and VP scanning was performed 30 s after the finish of the AP scanning.

### Data collection

Patient data were acquired from the electronic medical records. A total of 20 clinical, pathological, images and laboratory characteristics were evaluated referring to the World Health Organization and the American Joint Committee on Cancer (AJCC) TNM Staging System Manual, 8th Edition (24). Image characteristics were assessed by two radiologists with 8 and 10 years’ clinical experience, respectively. A consensus was reached when difference in opinion existed. The characteristics were classified as follows: (1) clinical characteristics: gender, age, abdominal pain, backache, pancreatitis, jaundice, operation method; (2) pathological characteristics: histological grade, duodenal invasion, surgical margin status, perineural invasion; (3) image characteristics: CT-reported tumor size, tumor location, clinical T stage, parenchymal atrophy, pancreatic duct dilatation, and common bile duct dilatation; (4) laboratory characteristics: carcino-embryonic antigen (CEA) level, carbohydrate antigen 19-9 (CA19-9) level, and total bilirubin (TBIL) level. Specific characteristics description can be referred in the Supplementary Material. Meanwhile, valuable characteristics were selected from above-mentioned characteristics using univariate and multivariable logistic regression analysis for clinical model building, except for that, we chose some feature, which is deemed meaningful in clinical experience for predicting tumor heterogeneity to further perfect this clinical model.

### Image segmentation and preprocessing

**Figure 2:**
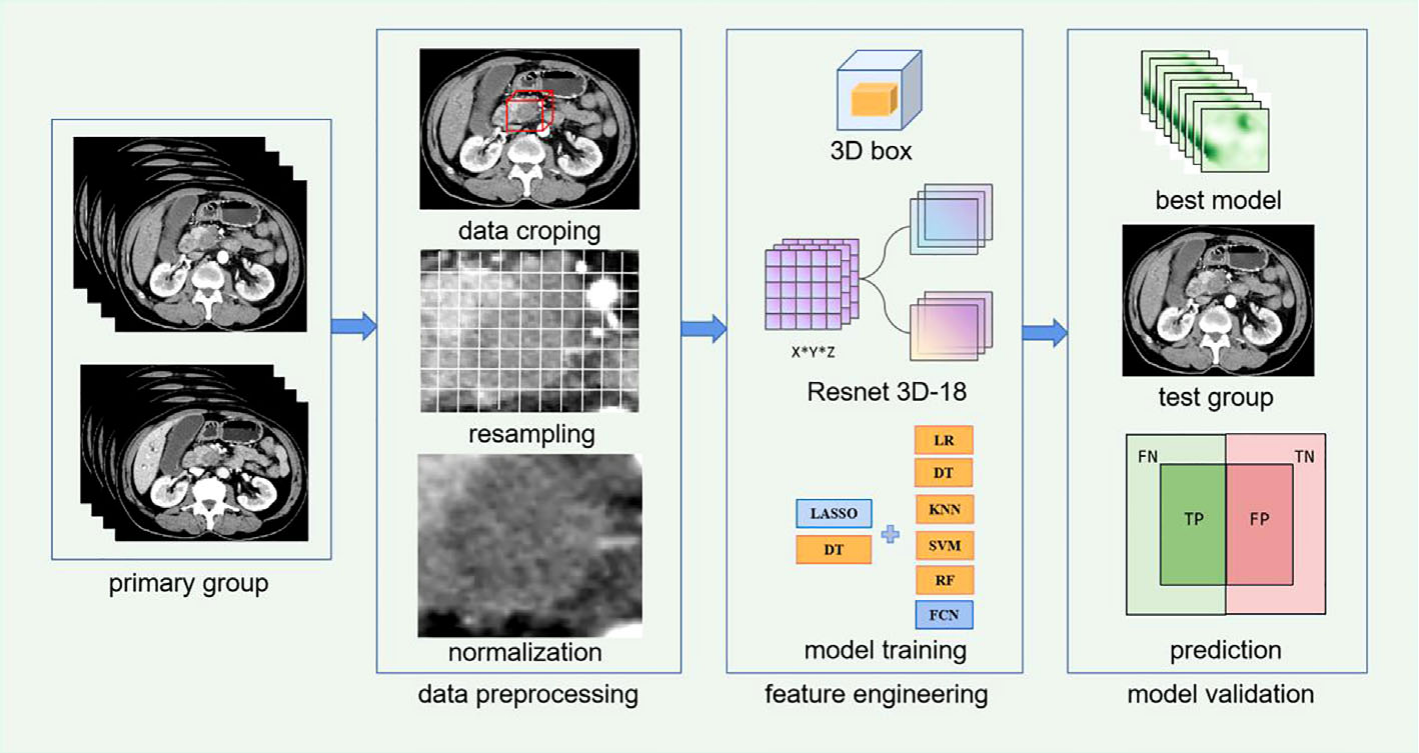
Workflow of Resnet 3D-18 model based on CECT for lymph node metastases (LNM) of patients with pancreatic ductal adenocarcinoma (PDAC).

In this study, traditional manual segmentation layer by layer is needless. We designed one 3D stereochemical box as the VOI. First, the tumor was localized by two experienced radiologist without precisely segmentation. One radiologist localized the lesion and another radiologist checked the accuracy of location. Second, this 3D box, which contained the complete tumor and slight peritumoral tissue along height-axis, width-axis, and depth-axis in every slice, is determined according to the location mark. Specifically, the original images could be recognized as a 3D array, and the largest peripheral box including the tumor is also a 3D array, and the index corresponding to each pixel of the tumor 3D box in the original images could be determined *via* computer language. The corresponding original images were cropped out according to the index for retaining tumor 3D box, and the specific demand of box size only need to retain whole tumor as much as possible because deep learning algorithm is more robust and strong than traditional machine learning method, the requirement of precise segmentation in deep learning is not as strict as traditional radiomics. This method is consistent with An’s study about the localization and segmentation technology (26). Third, after subtracting the periphery regions out of tumor 3D box, original 3D images and 3D segmentation masks (ground truth) were resampled to a specified resolution of 5*224*224, and images were processed to (0,1) using min-max normalization. A complete workflow is displayed in **Figure 2**.

### Deep learning model selection

Resnet as a celebrated CNN is proposed by Microsoft research. In 2015, Resnet won the image classification and object recognition competition in ImageNet Large Scale Visual Recognition Challenge (ILSVRC). The Resnet 18 represent 18 layers deep networks constructed by internal residual blocks, which were implemented by shortcut connection, thereby performing identity mapping and solving degradation problem caused by over-deeper layers. But Resnet 18, as one 2D CNN model, could only extract feature in single slice of 2D CT images without extracting the entirety in real 3D structure. Thus, Resnet 3D-18 was optimizing and upgrading on the basis of Resnet 18. Resnet 3D-18 could extract context features comprehensively and globally using automatic parameter learning, thereby avoiding losing stereoscopic information. Currently, Resnet 3D-18 is highly appreciated by its excellent learning performance and optimization ability, and increasingly applied in image segmentation, recognition, classification. Moreover, it has been reported that Resnet-3D model achieve better accuracy compared to 2D ones, and the deeper networks (34 layers) show little gain over 18 layer ones (33, 34), so the Resnet 3D-18 architecture was appropriate and selected for subsequent analysis.

### Deep learning feature extraction and selection

Pretrained Resnet 3D-18 model on the ImageNet database with the method of self-supervised learning was implemented for feature extraction (35, 36). In previous studies of deep learning neural networks (25, 27), end-to-end learning methods were regularly used without feature selection procedure; however, these features are not identically significant to the issues. In this experiment, multiple attempts in feature selection method were conducted, including decision tree (DT) and least absolute shrinkage and selection operator (LASSO) model. Ultimately, the LASSO model was selected, which is consistent with prior deep learning radiomics studies (37). All extracted features are fed into the LASSO model. The loss function of LASSO is calculated as below:


$$\text{Loss}=\frac{1}{2\text{N}}\sum_{\text{n}=1}^{\text{N}}∥\text{y}-\text{Xw}{∥}_{2}^{2}+\lambda^{*}|\left|w\right|{|}_{1}$$

Where N is the number of all samples, y represents the true label, and X represents feature, w represents weight of feature, and hyperparameter λ denotes penalty coefficient. Those features with non-zero weight were retained. The λ was searched in 0.005-0.02 using the traversal method. The larger the λ is, the stronger the effect of the regular term is, and more weights would be compressed to zero. The model error was calculated by putting the possible values into the model. The consequence of λ yielding the minimum error was selected as the optimal hyperparameter.

### DLR model construction

In this experiment, we have attempted six classifiers including logistic regression (LR), k-nearest neighbor (KNN), SVM, DT, random forest (RF), and fully connected neural network (FCN). Finally, the combination of LASSO+FCN achieving the best robustness and stability was selected, followed by the combination of DT + RF, which were easily influenced by randomness of the data set, thereby being excluded. Then, the features retained by LASSO were fed into FCN, which is a linear model to calculate the final probability score. The architecture of FCN was constructed by one hidden layer and one output layer with training epochs of 30. Limited Broyden-Fletcher-Goldfarb-Shanno (Lbfgs) optimizer was adopted to minimize the loss function—binary cross-entropy—which is calculated as follows:


$$\text{L}=\frac{1}{\text{N}}\sum_{\text{i}}{\text{L}}_{\text{i}}=\frac{1}{\text{N}}\sum_{\text{i}}-[{\text{y}}_{\text{i}}\cdot \text{log}({\text{p}}_{\text{i}})+(1-{\text{y}}_{\text{i}})\cdot \text{log}(1-{\text{p}}_{\text{i}})]\;$$

Where *y*_*i*_ represents true label of sample i, the positive sample is 1, and the negative sample is 0. *p*_*i*_  represents the probability of the sample predicted as a positive class. In hidden layer, neurons were set to 1,000, and the activation function used was rectified linear unit (Relu), which could make the output of some neurons be zero, thereby contributing to the sparsity of the network, decreasing the interdependence of parameters, and relieving the occurrence of over-fitting issues. In output layer, neurons were set to two, and the activation function was softmax function *via* mapping the values of the output layer to the 0-1 interval as a probability distribution where z represent values of the output layer. The larger the softmax value is, the better the model predicts. The formula of softmax is:


$$\text{Softmax}({\text{z}}_{\text{i}})=\frac{\text{exp}({\text{z}}_{\text{i}})}{{\text{Σ}}_{\text{j}}\text{exp}({\text{z}}_{\text{j}})}$$

The CNN training process included two aspects (backward propagation and forward propagation). First, five times 5-fold cross-validation was set for training model. The primary group was stochastically shuffled and divided into 5-fold averagely. In every time among five times, 1-fold was regarded as validation group successively, and the other 4 folds were regarded as training group in this order. Then, the images were fed into the network, and predictive result through forward propagation displayed in the network’s output layer. Simultaneously, the model parameters were updated and decided *via* backward propagation until achieving the minimum difference between the true label and predictive result. In addition, we used early stopping, which is a technique use to stop training before overfitting occurs. The use of early stopping can obtain the best generalization performance and prevent overfitting by intercepting the model with the best results in the whole process of model training. In total, three 3D CNN models including AP DLR model, VP DLR model, and AP+VP DLR model were separately built. The predictive ability of three CNN models was exhibited among every time in 5-fold cross-validation. The general prediction result was fused among these 5-fold groups by averaging the scores. The independent test group was employed to evaluate the model performance. It should be noted that no patients in the test group directly or indirectly participate in the model training process, thereby avoiding data leakage.

### Performance evaluation of DLR model

In our study, the following five quantitative indicators were calculated: the area under the curve (AUC), accuracy, precision, recall, and F1 score. Due to the imbalances between the LNM group and non-LNM group, we used the AUC as our principle evaluation indicator, followed by accuracy. Furthermore, the Radiomics score (Rad score) were calculated through a linear combination of features’ weighted coefficients. To make a comparison of diagnostic performance between the 3D CNN models with physician-level accuracy, CECT images of all 139 patients were respectively reviewed by two abdominal radiologists (senior radiologist and junior radiologist) following double-blind principle. The site of lymph node resection is consistent with that of images measurement. The accuracy, sensitivity, specificity, positive predictive value (PPV) and negative predictive value (NPV) were evaluated in performance of radiologists.

### Statistical analysis and experiment implementation

Continuous variables were analyzed using Student’s *t* test or the Mann–Whitney *U* test; Categorical variables were analyzed by chi-square test or Fisher’s exact test. Wilcoxon rank sum test was used to compare Rad scores in negative and positive groups. Delong test was applied to evaluate the discrimination ability among AUCs, and *P<*0.05 was regarded as statistically significant. Multivariable logistic regression analyses use the likelihood ratio test with Akaike’s information criterion as the stopping rule. All statistical tests were executed with SPSS software (version 25.0), R software (version 4.0.5), and Python software (version 3.8.0). In this study, the pre-processed and feature extraction approaches were conducted using SimpleITK, numpy, and scikit-learn package; the PyTorch 1.0 configuration was arranged to build the neural network. The training process was conducted on Ubuntu OS with an Intel Xeon E5 2687W V3, NVIDIA GeForce 1080ti GPU, and 16 × 8GB of RAM.

## Results

### Patient

A total of 139 patients (99 men, 40 women) were recruited for our study, and were partitioned into a primary group (n = 111) and a test group (n = 28). The LNM rates in every group were 35.1% (39/111) and 32.1% (9/28), respectively, and *P>*0.05 ensuring grouping consistency. In the primary group, histological grade, duodenum invasion, and CT-reported tumor size were notably different between non-LNM and LNM cohorts (**Table 1**). There were no significant differences in the test group. Some studies suggested that CA19-9 level is an independent predictive factor for LNM, and the higher CA19-9 levels indicating a worse patient’s condition (38). Thus, CA19-9 is enrolled for subsequent analysis. Univariate and multivariable logistic regression analysis demonstrated that histological grade, CT-reported tumor size, and CA19-9 were independent predictors of LNM in PDAC (**Table 2**). PDAC patients with LNM were more likely to have higher histological grade (OR,0.175; 95% CI, 0.061 to 0.499), larger CT-reported tumor size (OR, 1.182; 95% CI, 1.069 to 1.307), abnormal CA 199 level (OR, 4.139; 95% CI, 1.300 to 13.175).

**Table 1:** Baseline characteristics in the primary and test groups.

Characteristics	Primary group (n=111)		*P* value	Test group (n=28)		*P* value
	Non-LNM (n=72)	LNM (n=39)		Non-LNM (n=19)	LNM (n=9)
**Clinical characteristics**						
Age (y), mean ± SD	60.68 ± 9.96	62.11 ± 8.47	0.456	63.84 ± 8.46	59.56 ± 11.94	0.283
Gender, n (%)			0.585			0.609
Female	22 (30.6)	10 (25.6)		6 (31.6)	2 (22.2)	
Male	50 (69.4)	29 (74.4)		13 (68.4)	7 (77.8)	
Abdominal pain			0.255			0.439
Yes	40 (55.6)	26 (66.7)		12 (63.2)	7 (77.8)	
No	32 (44.4)	13 (33.3)		7 (36.8)	2 (22.2)	
Backache			0.307			0.483
Yes	17 (23.6)	6 (15.4)		4 (21.1)	3 (33.3)	
No	55 (76.4)	33 (84.6)		15 (78.9)	6 (66.7)	
Pancreatitis			0.314			0.159
Yes	8 (11.1)	7 (17.9)		7 (36.8)	1 (11.1)	
No	64 (88.9)	32 (82.1)		12 (63.2)	8 (88.9)	
Jaundice			0.263			0.926
Yes	9 (12.5)	8 (20.5)		6 (31.6)	3 (33.3)	
No	63 (87.5)	31 (79.5)		13 (68.4)	6 (66.7)	
Operation, n (%)			0.388			0.521
Pancreaticoduodenectomy	56 (77.8)	33 (84.6)		15 (78.9)	8 (88.9)	
Distal pancreatectomy	16 (22.2)	6 (15.4)		4 (21.1)	1 (11.1)	
**Pathological characteristics**						
Histological grade, n (%)			0.001*			0.505
Low-grade	55 (76.4)	18 (46.2)		11 (57.9)	4 (44.4)	
High-grade	17 (23.6)	21 (53.8)		8 (42.1)	5 (55.6)	
Duodenum Invasion, n (%)			0.001*			0.907
Negative	51 (70.8)	15 (38.5)		8 (42.1)	4 (44.4)	
Positive	21 (29.2)	24 (61.5)		11 (57.9)	5 (55.6)	
Surgical margin status, n (%)			0.196			0.175
Negative	69 (95.8)	39 (100)		18 (94.7)	7 (77.8)	
Positive	3 (4.2)	0 (0)		1 (5.3)	2 (22.2)	
Perineural invasion, n (%)			0.397			0.409
Negative	7 (9.7)	2 (5.1)		2 (10.5)	2 (22.2)	
Positive	65 (90.3)	37 (94.9)		17 (89.5)	7 (77.8)	
**Imaging characteristics**						
CT-reported tumor size (mm), mean ± SD	23.99 ± 9.62	29.03 ± 13.69	0.026*	29.68 ± 12.13	34.33 ± 6.70	0.295
Location, n (%)			0.639			0.726
Head and neck	58 (80.6)	33 (84.6)		16 (84.2)	8 (88.9)	
Body and tail	14 (19.4)	6 (15.4)		3 (15.8)	1 (11.1)	
T stage, n (%)			0.099			0.735
cT1	24 (33.3)	7 (17.9)		1 (5.3)	0 (0)	
cT2	41 (56.9)	25 (64.1)		15 (78.9)	7 (77.8)	
cT3-4	7 (9.7)	7 (17.)		3 (15.8)	2 (22.2)	
Parenchymal atrophy, n (%)			0.428			0.885
Yes	40 (56.3)	25 (64.1)		10 (52.6)	5 (55.6)	
No	31 (43.7)	14 (35.9)		9 (47.4)	4 (44.4)	
PD dilatation, n (%)			0.484			0.409
Yes	57 (79.2)	33 (84.6)		17 (89.5)	7 (77.8)	
No	15 (20.8)	6 (15.4)		2 (10.5)	2 (22.2)	
CBD dilatation, n (%)			0.158			0.815
Yes	46 (63.9)	30 (76.9)		14 (73.7)	7 (77.8)	
No	26 (36.1)	9 (23.1)		5 (26.3)	2 (22.2)	
**Laboratory characteristics**						
CA-199 level, n (%)			0.121			0.409
Normal	16 (22.2)	14 (35.9)		2 (10.5)	2 (22.2)	
Abnormal	56 (77.8)	25 (64.1)		17 (89.5)	7 (77.8)	
CEA level, n (%)			0.149			0.521
Normal	63 (87.5)	30 (76.9)		15 (78.9)	8 (88.9)	
Abnormal	9 (12.5)	9 (23.1)		4 (21.1)	1 (11.1)	
TBIL level, n (%)			0.312			0.439
Normal	33 (45.8)	14 (35.9)		7 (36.8)	2 (22.2)	
Abnormal	39 (54.2)	25 (64.1)		12 (63.2)	7 (77.8)	

* represents the p values that are smaller than 0.05. Categorical data are number of patients; data in parentheses are percentage.

PD, pancreatic duct; CBD, common bile duct; CA 19–9, carbohydrate antigen 19–9; CEA, carcino-embryonic antigen; TBIL, total bilirubin.

**Table 2:** Univariate and multivariable logistic regression analyses in selecting features.

Variable	Univariate analysis				Multivariate analysis			
	β	Wald	Odds ratio (95%CI)	*P*-value	β	Wald	Odds ratio (95%CI)	*P*-value
**Clinical characteristics**								
Age	-0.013	0.453	0.987 (0.951-1.025)	0.501				
Gender	-0.288	0.509	0.750 (0.340-1.653)	0.476				
Abdominal pain	-0.501	1.768	0.606 (0.290-1.268)	0.184				
Backache	0.262	0.347	1.300 (0.543-3.11)	0.556				
Pancreatitis	-0.013	0.001	0.987 (0.386-2.525)	0.978				
Jaundice	-0.410	0.849	0.664 (0.278-1.587)	0.357				
Operation	0.501	1.084	1.650 (0.643-4.235)	0.298				
**Pathological characteristics**								
Histological grade	-1.138	9.310	0.321 (0.154-0.666)	0.002*	-1.745	10.621	0.175 (0.061-0.499)	0.001*
Duodenum Invasion	-1.035	7.911	0.355 (0.173-0.731)	0.005*				
Surgical margin status	0.056	0.004	1.057 (0.187-5.992)	0.950				
Perineural invasion	-0.188	0.090	0.828 (0.241-2.843)	0.765				
**Imaging characteristics**								
CT-reported tumor size	0.048	8.172	1.050 (1.015-1.085)	0.004*	0.167	0.051	1.182 (1.069-1.307)	0.001*
Location	0.466	1.036	1.594 (0.649-3.913)	0.309				
Clinical T stage	-1.168	3.460	0.311 (0.091-1.065)	0.063				
Parenchymal atrophy	-0.288	0.618	0.750 (0.366-1.536)	0.432				
PD dilatation	-0.139	0.086	0.871 (0.346-2.194)	0.769				
CBD dilatation	-0.553	1.831	0.575 (0.258-1.281)	0.176				
**Laboratory characteristics**								
CA-199 level	-0.707	3.066	2.028 (0.919-4.474)	0.080	1.420	5.782	4.139 (1.300-13.175)	0.016*
CEA level	-0.457	0.966	0.633 (0.255-1.575)	0.326				
TBIL level	-0.450	1.465	0.638 (0.308-1.322)	0.226				

* represents the p values that are smaller than 0.05. CI confidence interval.

PD, pancreatic duct; CBD, common bile duct; CA 19–9, carbohydrate antigen 19–9; CEA, carcino-embryonic antigen; TBIL, total bilirubin.

### Deep learning features selection and construction

In this study, we attempted experiments comparing DLR model with feature selection procedure or without feature selection procedure. Without feature selection, the learning curves were overfitting in the training group, and were not converged in validation group and test group in all three models. However, learning curves improved significantly and reached perfect fitting with feature selection. The training curves of three models could be found in **Supplementary Figure S1**. This finding suggested that feature selection may be an important method in deep learning researches, especially in small data sets. Adding feature selection procedure might achieve better performance.

**Figure 3:**
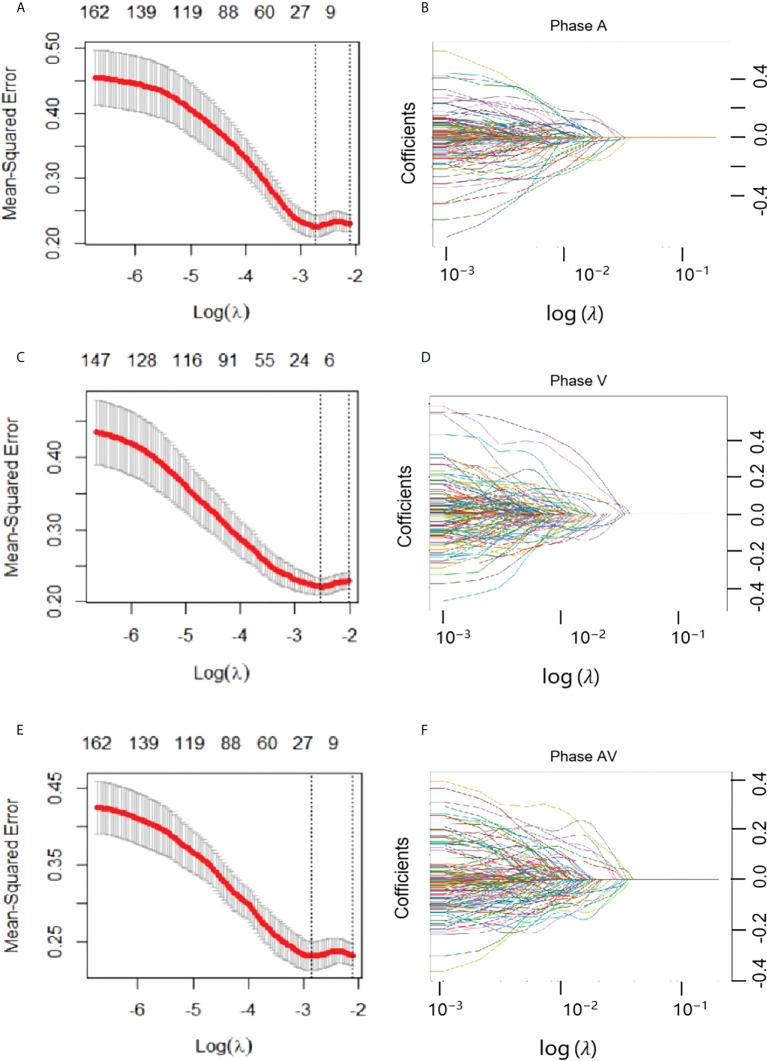
Feature selection with the least absolute shrinkage and selection operator (LASSO) model. **(A, B)** represented AP model, and **(C, D)** represented VP model, and **(E, F)** represented AP+VP model. **(A, C, E)** The LASSO model’s tuning parameter (λ) selection used five-fold cross-validation *via* minimum criterion. The vertical lines indicate the optimal value of the LASSO tuning parameter (λ). **(D, E, F)** LASSO coefficient profile plot with different log (λ) was displayed.

**Figure 4:**
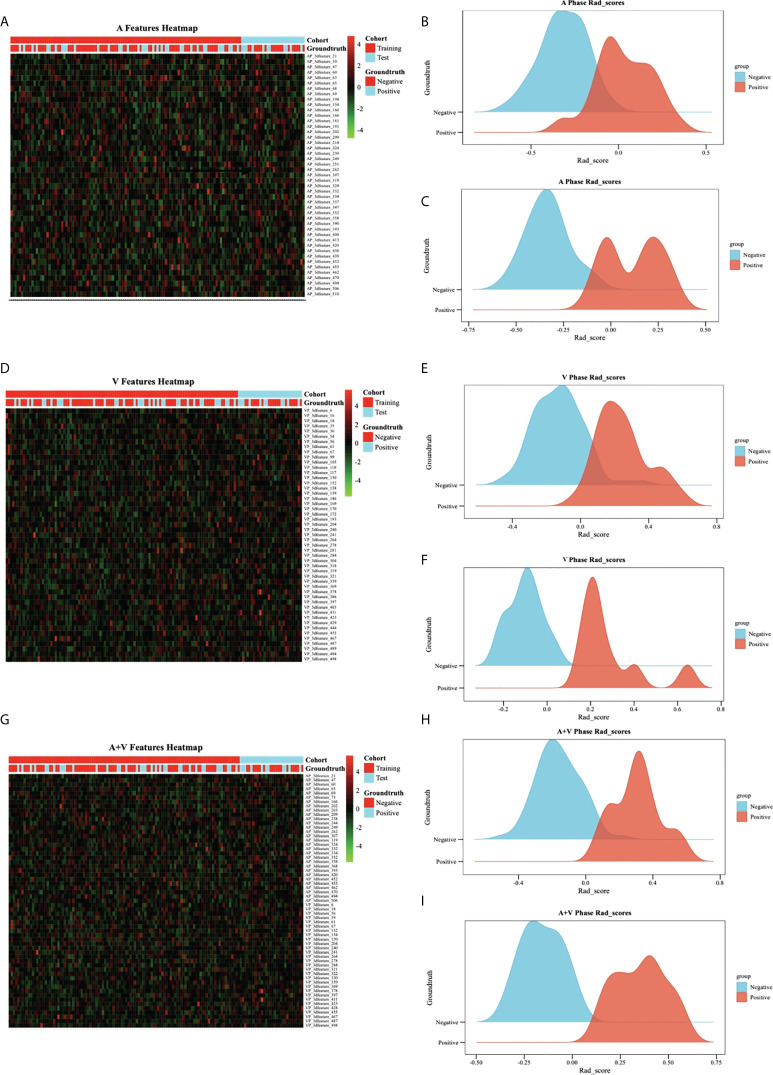
Feature heatmap and Rad score in different models.**(A–C)** represented AP model, and **(D–F)** represented VP model, and **(G–I)** represented AP+VP model. The heatmap is grouped according to primary group and test group. Each row corresponds to one deep learning feature, and each column corresponds to one patient. The ridgeline plot of the Rad scores in the LNM cohort (blue part) and the non-LNM cohort (orange part) showed significant difference between the two cohorts (all *P*>0.05).

The Resnet 3D-18 model separately extracted 512 deep learning features from AP and VP. Then, the most representative and significative characteristics were retained by LASSO model with λ setting as 0.01 (**Figure 3**). A total of 45, 49, and 59 features were selected from AP model, VP model, and fusion of AP+VP model, respectively. Indeed, more features were retained due to a large base of total deep learning features, which is greatly increased by the hidden layer structure of deep neural networks. These features with non-zero weights were ultimately assigned to construct DLR model. The feature heatmap and Rad score were plotted in **Figure 4**, and specific Rad score computational formula, including corresponding coefficients, was in Supplementary Material. No matter in any of the three 3D CNN models, significant difference was observed in the Rad score between patients in LNM and non-LNM groups (all *P*< 0.01).

### Performance evaluation of DLR models, clinical model, and radiologists

**Figure 5:**
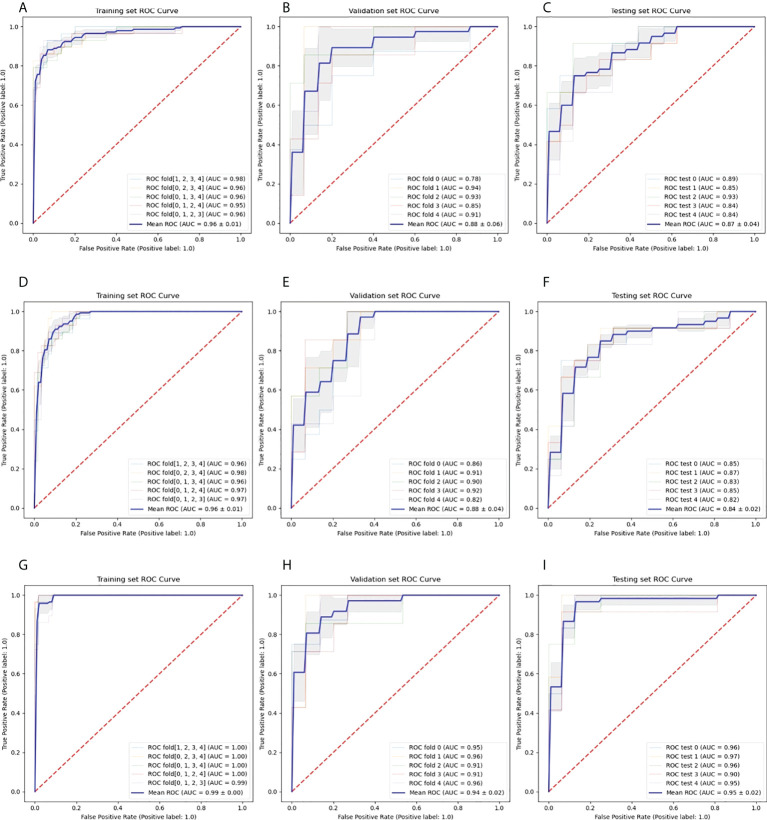
Roc curves of 3D CNN models.**(A–C)** represented AP model in training **(A)**, validation **(B)**, and test **(C)** groups, and **(D–F)** represented VP model in training **(D)**, validation **(E)**, and test **(F)** groups, and **(G–I)** represented AP+VP model in training **(G)**, validation **(H)**, and test **(I)** groups. Every figure demonstrated model performance under 5-fold cross-validation.

The performance of different 3D CNN models are showed in **Table 3** and **Figure 5**. Overall speaking, the AP + VP DLR model reached the optimal ability for identifying LNM in PDAC with an AUC of 0.995 (95% CI: 0.989-1.00) and an accuracy of 0.969 in the training group; an AUC of 0.940 (95% CI:0.910-0.971) and an accuracy of 0.883 in the validation group; an AUC of 0.949 (95% CI:0.914-0.984) and an accuracy of 0.836 in the test group, followed by AP DLR model with an AUC of 0.962 (95% CI: 0.951-0.972) and an accuracy of 0.926 in the training group; an AUC of 0.884 (95% CI: 0.800-0.968) and an accuracy of 0.821 in the validation group; an AUC of 0.872 (95% CI: 0.823-0.921) and an accuracy of 0.736 in the test group; and the VP DLR model reached an AUC of 0.967 (95% CI:0.955-0.979) and an accuracy of 0.903 in the training group; an AUC of 0.884 (95% CI:0.829-0.938) and an accuracy of 0.784 in the validation group; an AUC of 0.844 (95% CI:0.820-0.867) and an accuracy of 0.764 in the test group. The AP model and VP model achieved similar performance, while both were not as good as AP+VP model.

**Table 3:** The performance of different CNN models.

Models	Cohorts	AUC (95%CI)	Accuracy	Precision	Recall	F1 score
AP DLR	Train	0.962 (0.951-0.972)	0.926	0.924	0.840	0.879
	Validation	0.884 (0.800-0.968)	0.821	0.756	0.675	0.701
	Test	0.872 (0.823-0.921)	0.736	0.938	0.417	0.565
VP DLR	Train	0.967 (0.955-0.979)	0.903	0.845	0.861	0.852
	Validation	0.884 (0.829-0.938)	0.784	0.726	0.607	0.625
	Test	0.844 (0.820-0.867)	0.764	0.797	0.600	0.678
AP + VP DLR	Train	0.995 (0.989-1.00)	0.969	0.952	0.952	0.952
	Validation	0.940 (0.910-0.971)	0.883	0.896	0.750	0.810
	Test	0.949 (0.914-0.984)	0.836	0.909	0.683	0.776

AP, arterial phase; VP, venous phase; CI, confidence interval.

**Figure 6:**
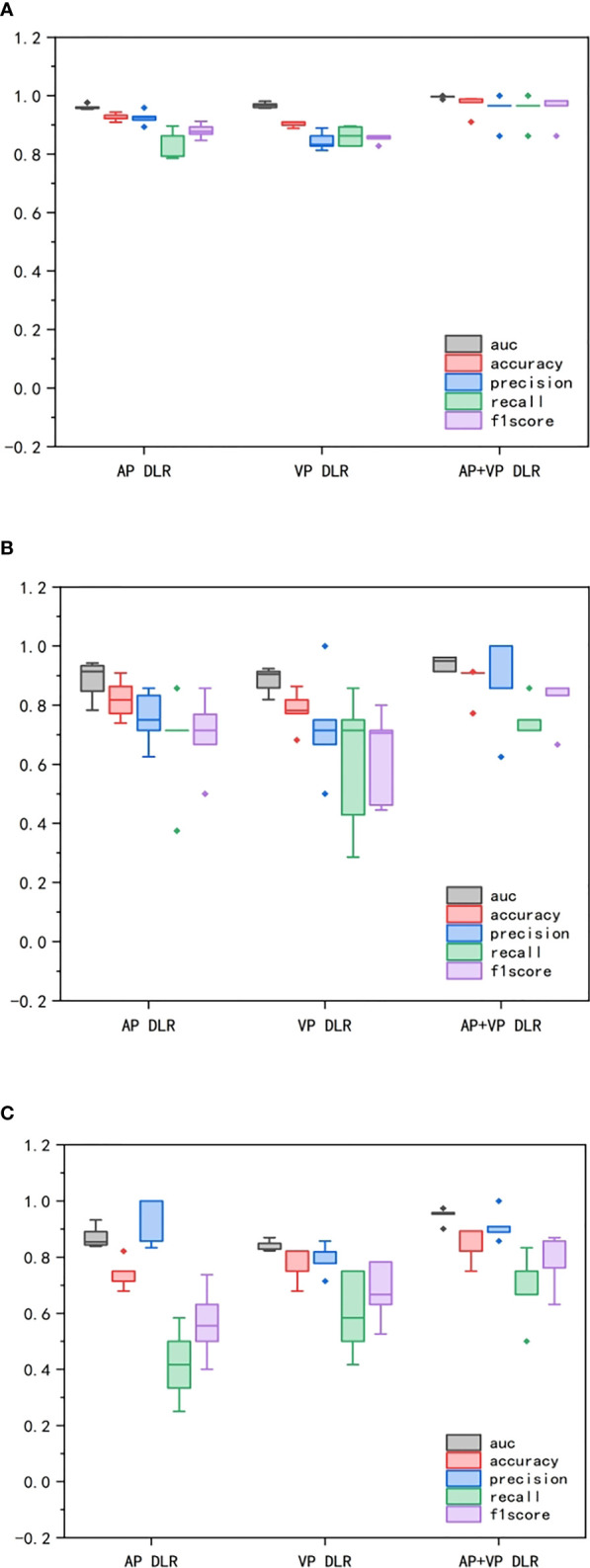
Comparison of performance among CNN models in different groups. **(A)** represented training group. **(B)** represented validation group. **(C)** represented test group. The box-and-whisker plots demonstrated the differences in AP, VP, AP+VP models among AUC, accuracy, precision, recall, and F1 score.

Moreover, we could observed that all models reached decent outcome with most AUCs ranging from 0.92-1.00 in every fold among 5-fold cross-validation. The ROC curves including every fold curve and average curve in training group and test group in all models kept stable and slight wobble. The performance comparison of the different 3D CNN models in training, validation, and test group were displayed in **Figure 6**. No matter in training, validation, and test groups, all statistical indicators in AP+VP model were the highest than that of other models, except in test group, precision is lower in AP+VP model than AP model. Delong test demonstrated no significant difference was observed in test group with AUCs ranging from 0.844 to 0.949 (all *P*>0.05), denoting the robustness and consistency of AP, VP, and AP+VP models.

**Figure 7:**
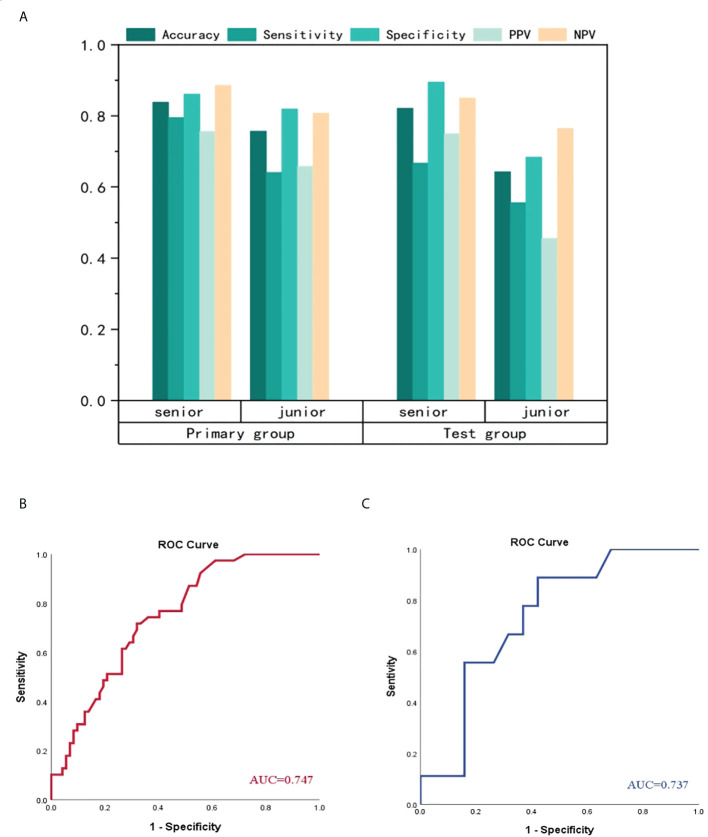
Comparison of performance among radiologists and clinical model **(A)** represented manifestation of senior radiologist and junior radiologist. No matter in primary group or test group, the senior radiologist performed better than junior radiologist. The clinical model achieved ordinary performance in primary group **(B)** and test group **(C)**, which is the worst among CNN models, radiologists and clinical model.

The performance of radiologists and clinical model were showed in **Table 4** and **Figure 7**. In primary group, the accuracy of senior radiologist was 0.838, which was slightly lower than that of AP DLR model and VP DLR model with an accuracy of 0.874 and 0.844, respectively. In test group, the accuracy of senior radiologist was 0.821, which is also higher than that of AP DLR model and VP DLR model with an accuracy of 0.736 and 0.764, respectively. However, the predictive ability of senior radiologist was lower than that of AP+VP DLR model in both primary group and test group. The predictive ability of junior radiologist was lower than that of AP DLR model, VP DLR model, and AP+VP DLR model in primary group and test group. A clinical model was constructed by concatenating histological grade, CT-reported tumor size, and CA19-9 level. The clinical model achieved an AUC of 0.747 (95% CI:0.657-0.837) in primary group and an AUC of 0.737 (95% CI:0.549-0.925) in test group (**Figure 6**), which is lower than all 3D CNN models and radiologists.

**Table 4:** The performance of radiologists and clinical model.

Radiologist/Clinical	Group	AUC(95%CI)	Accuracy	Sensitivity	Specificity	PPV	NPV
Senior	Primary	–	0.838	0.795	0.861	0.756	0.886
	Test	–	0.821	0.667	0.895	0.75	0.85
Junior	Primary	–	0.757	0.641	0.819	0.658	0.808
	Test	–	0.643	0.556	0.684	0.455	0.765
Clinical	Primary	0.747 (0.657-0.837)	0.685	0.847	0.385	0.718	0.577
	Test	0.737 (0.549-0.925)	0.714	0.842	0.444	0.762	0.571

PPV, positive predictive value; NPV, negative predictive value; CI, confidence interval.

## Discussion

In this study, we designed and validated 3D CNN models based on 3D VOI segmentation technology for constructing different DLR strategy. Ultimately, the AP+VP DLR model achieved excellent repeatability and robustness with an AUC of 0.995 in training group, 0.940 in validation group and 0.949 in an independent test group, which is better than other 3D CNN models, clinical model, and radiologists. Therefore, this model could serve as an outstanding assistant tool in clinical decision-making and alleviating costly manual work in traditional machine learning researches.

Previous report had pointed out that 3D volumetric data are required in future studies for comparing and improving the performance of the 2D ROI-based texture metrics (39). As yet, it is a clinical challenge to differentiate LNM in PDAC non-invasively. Traditional radiomics method constructed models on the basis of the images by intelligent calculation to acquire relevant phenotypic characteristics, and has been widely used for researches on pancreatic diseases. Previous radiomics studies predicting LNM in PDAC mostly used traditional machine learning methods or texture analysis (28–32), which needed time-consuming manual segmentation of tumor boundary and extracted relatively low-level features, and actually were regarded as one statistical analysis without application of advanced algorithm. Like Bian’s radiomics study for predicting LNM in PDAC (29), the predictive outcome achieved an AUC of 0.75 in training group and an AUC of 0.81 in validation group. This article just used hand-crafted features for analysis without machine learning model. The similar traditional radiomics approach was executed in Gao’s study (28), which used Rad score for differentiating difference between LNM and non-LNM groups, with an AUC of 0.90 in training group and 0.89 in validation group. Liang (31) also reported a nomogram integrating Rad score and CT for visualization in identifying LNM in PDAC, with an AUC of 0.80 in primary cohort and 0.78 in validation cohort. In general, these studies used equal procedures in feature extraction and model building, not only costing physician resources but also generating modest results without progress and improvement continuously. Thus, we have reasons to believe that this 3D CNN architecture could obtain better outcome due to the network’s strong adaptability and generalization ability.

In common neural network models, there existed more or less issues like messages loss when transmitting messages. We chose the state-of-the-art network (Resnet) to solve this problem by straightforwardly bypassing the input information to the output, guarding the integrity of the information. The entire network merely needs to study the difference between the input and output, reducing the learning targets and difficulties. In addition, this 3D volumetric network architecture, which could obtain context from adjacent slices for grasping richer boundary information about the pancreas (40). As expected, the performance of 3D CNN network is better than 2D network. Beyond that, most radiomics studies only used one scanning phase for the purpose of convenience and selecting one phase, which displayed clearer lesion boundary (26, 41, 42). Like An’s study (26), which also used one 2D ROI-based 2D CNN algorithm with only one venous phase for predicting LNM in PDAC, whereas the best model integrating multiple radiomics model with clinical model achieved an AUC of 0.90 in validation group and 0.92 in test group. In our study, the AP+VP DLR model outperformed other one-contrast prediction models (AP DLR model, VP DLR model). In general, the Resnet 3D-18 AP+VP model in our study has achieved the best predictive performance than all previous published radiomics studies in differentiation of LNM in PDAC (28–32).

Our study demonstrated that low-grade (well+moderately differentiated) group was commonly observed in non-LNM group and larger tumor size was easily observed in LNM group, which is consistent with the results of Li and Liang (30, 31), indicating that LNM patients had higher invasiveness and poorer prognosis. In general, the clinical model combined the screened variables achieved ordinary performance. In view of the histological grade is obtained from post-operatively pathological examination, the model’s predictive performance will be further decreased after removing histological grade. In our study, radiologists reached decent performance *via* visual images evaluation, not only the minor axis of lymph node was measured, but also inherent structure and enhancement pattern was observed, thereby a final decision was made. And the predictive difference between senior radiologist and junior radiologist demonstrating visual evaluation is subjective and easily affected by radiologists’ clinical experience, resulting in the instability and inaccuracy of radiological report and bringing about the corresponding influence of clinical treatment strategy. In addition, the Resnet 3D-18, indeed, demonstrated greater accuracy and precision than the radiologists with 10–30 years of clinical experience, which denoting that artificial intelligence, indeed, could help clinical physicians.

This study denoted that the Restnet 3D-18 model enabled the segmentation procedure, saving time and energy. To our knowledge, this is the first study applying the 3D CNN model and 3D VOI-based strategy for predicting LNM in PDAC. It provided solid and stable evidence that the 3D CNN strategy offered an novel perspective and outperformed many traditional radiomics studies in model performance and generalization ability. This approach hold a promise of being a practical assistant tool in clinical practice.

This study still had some limitations. First, our sample size is relatively small and is derived from one single center. Enrolling a larger sample size and conducting multicenter study to further confirm the predictive ability are essential. Second, a potential selection bias existed due to the patients never suffering radical excision were excluded, which resulting in the imbalance of LNM group and non-LNM group. Third, we did not evaluate the delayed phase. Further prospective studies will investigate the value of delayed phase or select more advanced imaging technology such as dual-energy CT.

## Conclusion

In general, our study designed and validated 3D CNN model (Resnet 3D-18) with 3D VOI-based strategy based on multiphasic CECT images to differentiate LNM in PDAC patients. The AP+VP DLR model demonstrated the optimal predictive performance that is capable of further assisting in precision medicine and improving diagnostic performance.

## Data availability statement

The original contributions presented in the study are included in the article/**Supplementary Material**. Further inquiries can be directed to the corresponding author.

## Ethics statement

Written informed consent was not obtained from the individual(s) for the publication of any potentially identifiable images or data included in this article.

## Author contributions

HFL and YBL designed the study. HFL collected and assembled all data. HFL, JJY, and JYY performed data analysis. HFL wrote the manuscript. YBL, YML, and HL revised the manuscript. All authors contributed to the article and approved the final submitted version.

## Conflict of interest

The authors declare that the research was conducted in the absence of any commercial or financial relationships that could be construed as a potential conflict of interest.

## Publisher’s note

All claims expressed in this article are solely those of the authors and do not necessarily represent those of their affiliated organizations, or those of the publisher, the editors and the reviewers. Any product that may be evaluated in this article, or claim that may be made by its manufacturer, is not guaranteed or endorsed by the publisher.
