# Supplementary material for: One 3D VOI-based deep learning radiomics strategy, clinical model and radiologists for predicting lymph node metastases in pancreatic ductal adenocarcinoma based on multiphasic contrast-enhanced computer tomography
Source: Front Oncol. 2022 Sep 9;12:990156. doi: 10.3389/fonc.2022.990156 (PMC9500296; doi:10.3389/fonc.2022.990156)
Supplement: Supplementary file 1 [file DataSheet_1.docx]

Supplementary Material

**The definitions of the clinical features are listed as follows:**

1. clinical characteristics were recorded from [electronic](javascript:;) [medical](javascript:;) [record](javascript:;) including gender, age, [abdominal](javascript:;) [pain](javascript:;), backache, history of pancreatitis, jaundice, operation method
2. pathological characteristics were recorded from pathological report including duodenal invasion, surgical margin status and perineural invasion. Histological grade were set as low-grade and high-grade group in accordance with the 2019 WHO classification of tumors of the digestive system [1]; well differentiated and moderately differentiated cases were included in the low-grade group and poorly differentiated cases were set as the high-grade group.
3. imaging characteristics

1.CT-reported tumour size(mm): the maximum tumour diameter in cross section

2.tumour location: head and neck, body and tail

3.clinical T stage was assessed using CT-reported tumour size according to the AJCC TNM Staging System Manual, 8th Edition.

4.parenchyma in comparison to both the head- and tail-side parenchyma, showing a cave-in, slim, or slit-like appearance[2].

5.pancreatic duct dilatation: we define pancreatic duct dilatation as >3 mm

6.common bile duct dilatation: we define common bile duct dilatation as >10 mm

1. laboratory characteristics:

1.[carcino-embryonic](javascript:;) [antigen](javascript:;) (CEA) level: abnormal CEA as > 5 ng/ml

2.carbohydrate antigen 19-9 (CA19-9) level : abnormal CA199 > 37 U/ml

3.total bilirubin (TBIL) level: abnormal TBIL level as > 20.5μmol/L

Reference:

[1] Nagtegaal I, Odze R, Klimstra D. (2020) The 2019 WHO classification of tumours of the digestive system. Histopathology, 76(2),182–188. [doi:10.1111/his.13975](https://doi.org/10.1111/his.13975)

[2] Prokesch, R. W., Chow, L. C., Beaulieu, C. F., Bammer, R., & Jeffrey, R. B., Jr (2002). Isoattenuating pancreatic adenocarcinoma at multi-detector row CT: secondary signs. Radiology, 224(3), 764–768. <https://doi.org/10.1148/radiol.2243011284>

[3] Nakahodo J., Kikuyama M., Nojiri S., Chiba K., Yoshimoto K., Kamisawa T., Horiguchi S.I., Honda G. Focal Parenchymal Atrophy of Pancreas: An Important Sign of Underlying High-Grade Pancreatic Intraepithelial Neoplasia Without Invasive Carcinoma, i.e., Carcinoma in Situ. Pancreatology. 2020;20:1689–1697. doi: 10.1016/j.pan.2020.09.020.

**Calculation of Radiomics Scores in different models**

**Radiomics_score(AP)**=-0.162AP_3dfeature_21 - 0.039AP_3dfeature_30 + 0.1838AP_3dfeature_47 - 0.04AP_3dfeature_60 + 0.0217AP_3dfeature_63 + 0.034AP_3dfeature_65 + 0.0031AP_3dfeature_68 + 0.026AP_3dfeature_69 + 0.0221AP_3dfeature_104 - 0.032AP_3dfeature_154 + 0.0934AP_3dfeature_160 + 0.0588AP_3dfeature_166 - 0.034AP_3dfeature_181 + 0.0419AP_3dfeature_191 + 0.1319AP_3dfeature_202 - 0.004AP_3dfeature_209 - 0.011AP_3dfeature_214 - 0.142AP_3dfeature_224 + 0.028AP_3dfeature_230 - 0.253AP_3dfeature_249 + 0.0067AP_3dfeature_251 - 0.231AP_3dfeature_262 - 0.025AP_3dfeature_307 + 0.1327AP_3dfeature_319 + 0.1394AP_3dfeature_324 - 0.107AP_3dfeature_332 + 0.0995AP_3dfeature_334 + 0.1424AP_3dfeature_337 - 0.037AP_3dfeature_347 - 0.1AP_3dfeature_352 - 0.102AP_3dfeature_358 + 0.0313AP_3dfeature_390 - 0.221AP_3dfeature_393 + 0.0916AP_3dfeature_404 - 0.009AP_3dfeature_413 + 0.0381AP_3dfeature_420 - 0.007AP_3dfeature_438 - 0.01AP_3dfeature_439 + 0.0208AP_3dfeature_452 - 0.136AP_3dfeature_453 - 0.075AP_3dfeature_462 + 0.076AP_3dfeature_470 - 0.044AP_3dfeature_494 + 0.1242AP_3dfeature_506 - 5.387AP_3dfeature_510

**Radiomics_score(VP)**=-0.044*VP_3dfeature_6-0.020*VP_3dfeature_16-0.078*VP_3dfeature_18+0.0275*VP_3dfeature_35-0.059*VP_3dfeature_36-0.033*VP_3dfeature_54+0.3162*VP_3dfeature_56+0.0810*VP_3dfeature_61-0.019*VP_3dfeature_67-0.052*VP_3dfeature_99-0.014*VP_3dfeature_105-0.066*VP_3dfeature_110+0.0100*VP_3dfeature_117+0.0701*VP_3dfeature_130+0.0158*VP_3dfeature_132+0.0701*VP_3dfeature_138+0.0287*VP_3dfeature_139-0.005*VP_3dfeature_146+0.1323*VP_3dfeature_169-0.071*VP_3dfeature_170-0.098*VP_3dfeature_172-0.005*VP_3dfeature_193+0.0992*VP_3dfeature_204+0.1244*VP_3dfeature_240-0.129*VP_3dfeature_241-0.044*VP_3dfeature_264-0.071*VP_3dfeature_278+0.0300*VP_3dfeature_281-0.151*VP_3dfeature_284+0.0019*VP_3dfeature_304-0.017*VP_3dfeature_318-0.025*VP_3dfeature_319+0.0030*VP_3dfeature_321-0.125*VP_3dfeature_339+0.0320*VP_3dfeature_369+0.0135*VP_3dfeature_378-0.037*VP_3dfeature_386-0.127*VP_3dfeature_397+0.0044*VP_3dfeature_403+0.1200*VP_3dfeature_411-0.156*VP_3dfeature_423+0.0585*VP_3dfeature_429+0.0270*VP_3dfeature_444+0.3877*VP_3dfeature_455-0.076*VP_3dfeature_467-0.043*VP_3dfeature_487+0.0423*VP_3dfeature_489-0.037*VP_3dfeature_494-0.083*VP_3dfeature_498

**Radiomics_score(AP+VP)**=-0.093*AP_3dfeature_21+0.1648*AP_3dfeature_47-0.042*AP_3dfeature_60+0.0339*AP_3dfeature_65+0.0599*AP_3dfeature_69+0.0206*AP_3dfeature_73+0.0173*AP_3dfeature_166+0.0946*AP_3dfeature_202+0.0339*AP_3dfeature_203-0.069*AP_3dfeature_209-0.013*AP_3dfeature_238-0.101*AP_3dfeature_244-0.146*AP_3dfeature_249-0.109*AP_3dfeature_262-0.058*AP_3dfeature_307+0.1036*AP_3dfeature_319+0.1537*AP_3dfeature_324-0.052*AP_3dfeature_332+0.0867*AP_3dfeature_334-0.091*AP_3dfeature_352-0.122*AP_3dfeature_358+0.0246*AP_3dfeature_368-0.041*AP_3dfeature_393+0.0535*AP_3dfeature_420+0.0014*AP_3dfeature_452-0.052*AP_3dfeature_453-0.043*AP_3dfeature_462+0.0010*AP_3dfeature_470-0.036*AP_3dfeature_494+0.1477*AP_3dfeature_506-0.016*VP_3dfeature_6-0.016*VP_3dfeature_18+0.2733*VP_3dfeature_56+0.0057*VP_3dfeature_59+0.0508*VP_3dfeature_61-0.058*VP_3dfeature_67+0.0289*VP_3dfeature_132+0.0437*VP_3dfeature_134-0.092*VP_3dfeature_170+0.0987*VP_3dfeature_204+0.1238*VP_3dfeature_240-0.075*VP_3dfeature_241-0.071*VP_3dfeature_264-0.027*VP_3dfeature_278-0.151*VP_3dfeature_284+0.0253*VP_3dfeature_321-0.048*VP_3dfeature_322+0.0361*VP_3dfeature_330-0.064*VP_3dfeature_339+0.0142*VP_3dfeature_369+0.0260*VP_3dfeature_378-0.131*VP_3dfeature_397+0.0934*VP_3dfeature_411-0.063*VP_3dfeature_423-0.001*VP_3dfeature_428+0.2015*VP_3dfeature_455-0.053*VP_3dfeature_467-0.004*VP_3dfeature_487-0.047*VP_3dfeature_498


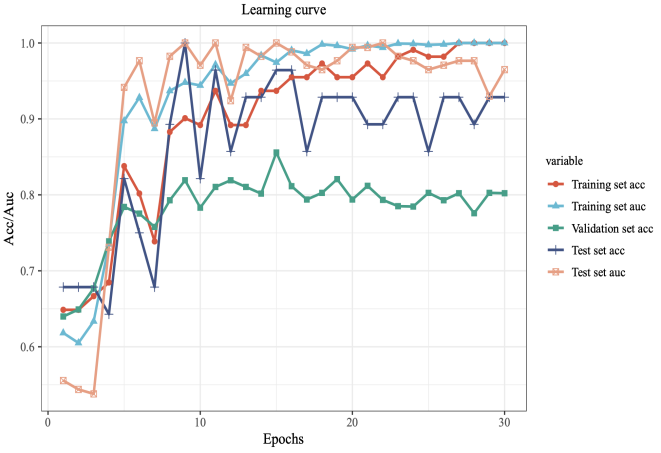

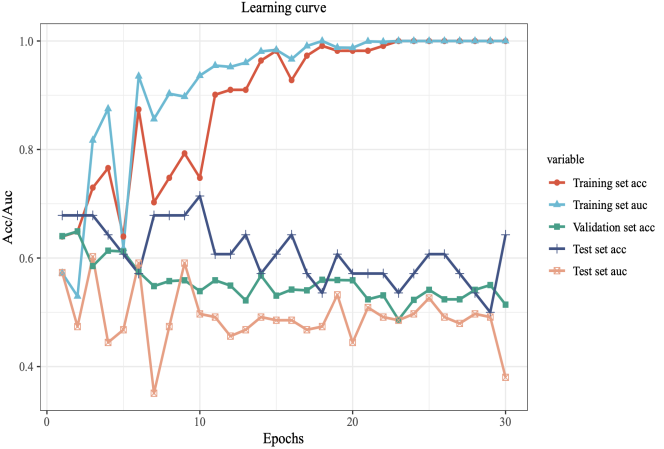

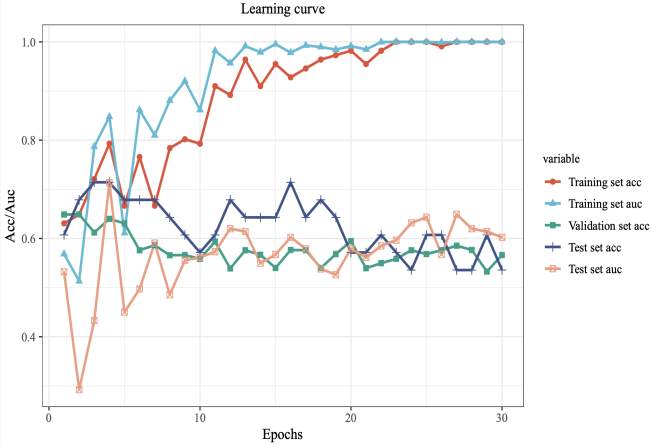

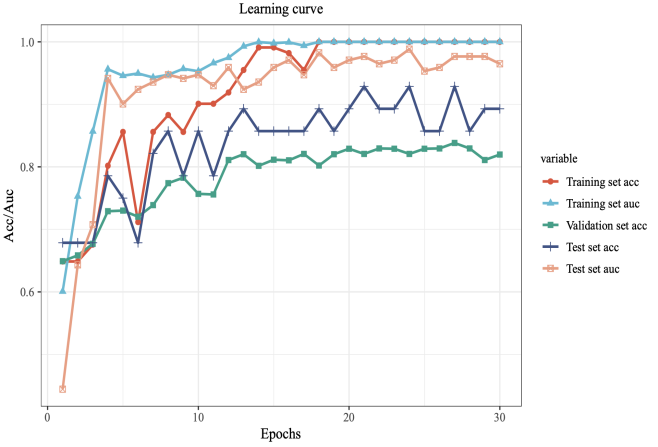

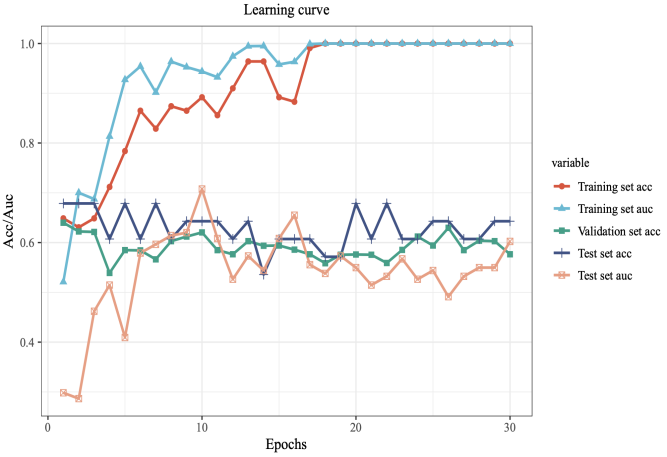

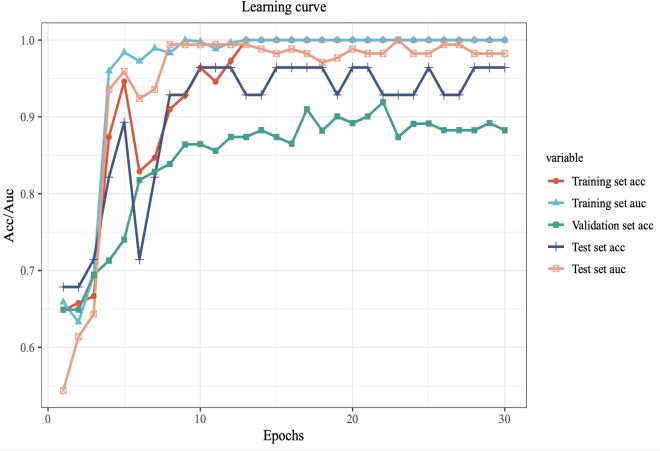


B

A

C

D

E

F

**Fig. S1 Comparison of learning curves between with feature selection and without feature selection.**

A.C.E respectively represent learning curves in AP model, VP model, AP+VP model without feature selection. The figures demonstrated training group was overfitting, whereas validation group and test group couldn’t converge. B.D.F respectively represent learning curves in AP model, VP model, AP+VP model with feature selection. The figures demonstrated learning curves reached perfect fitting in training, validation and test group.
